# Synthesis and characterization of homogeneous (U,Am)O_2_ and (U,Pu,Am)O_2_ nanopowders†

**DOI:** 10.1039/d2ce00527a

**Published:** 2022-08-31

**Authors:** Jean-François Vigier, Daniel Freis, Olaf Walter, Oliver Dieste Blanco, Daniel Bouëxière, Evelyn Zuleger, Natalia Palina, Tonya Vitova, Rudy J. M. Konings, Karin Popa

**Affiliations:** European Commission, Joint Research Centre (JRC) Karlsruhe Germany jean-francois.vigier@ec.europa.eu karin.popa@ec.europa.eu; Institute for Nuclear Waste Disposal (INE), Karlsruhe Institute of Technology P.O. 3640 D-76021 Karlsruhe Germany

## Abstract

This paper details the first dedicated production of homogeneous nanocrystalline particles of mixed actinide oxide solid solutions containing americium. The target compositions were U_0.75_Pu_0.20_Am_0.05_O_2_, U_0.90_Am_0.10_O_2_ and U_0.80_Am_0.20_O_2_. After successful hydrothermal synthesis and chemical characterisation, the nanocrystals were sintered and their structure and behaviour under self-irradiation were studied by powder XRD. Cationic charge distribution of the as-prepared nanocrystalline and sintered U_0.80_Am_0.20_O_2_ materials was investigated applying U M_4_ and Am M_5_ edge high energy resolution XANES (HR-XANES). Typical oxidation states detected for the cations are U(iv)/U(v) and Am(iii)/Am(iv). The measured crystallographic swelling was systematically smaller for the as-synthesised nanoparticles than the sintered products. For sintered pellets, the maximal volumetric swelling was about 0.8% at saturation, in line with literature data for PuO_2_, AmO_2_, (U,Pu)O_2_ or (U,Am)O_2_.

## Abstract 2

Sinterable homogeneous nanoparticles of [U,(Pu),Am]O_2_ are produced hydrothermally from corresponding oxalates. The uranium and americium oxidation states are diverse in both as-synthesized nanopowders and sintered materials.

## Graphical abstract

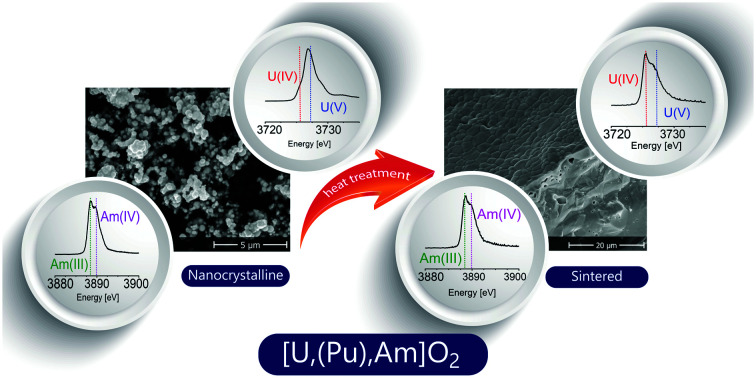

## 1. Introduction

One of the sustainability goals for nuclear reactors of the fourth generation (Gen-IV), as defined by the Generation IV International Forum (GIF), is the minimisation of the nuclear waste and the reduction of the long-term stewardship burden.^1^ Since plutonium and the minor actinides (MA) are largely responsible for the long-term radiotoxicity of the spent nuclear fuel,^2,3^ their separation and transmutation into short-lived isotopes in fast reactor systems can significantly contribute to this goal.^4^ The reuse of fissile isotopes from nuclear waste in the form of mixed oxide fuel is an integral part of the nuclear energy strategy in some EU member states and contributes to the sustainable usage of nuclear material resources. Similarly, the recycling of MA could lead to a further reduction of the radiotoxic inventory to be placed in the final deposits.^5^

The GIF sodium fast reactor advanced fuel project (SFR AF) targets at the development of minor actinide bearing fuels for irradiation in future SFR.^6^ Within the frame of this project, the Joint Research Centre investigates the performance of such fuels but also safe and reliable preparation routes. The preparation of MOX fuels containing MA requires energy and manpower, the workers then being exposed to radiation. Therefore, there is a need for development of processes that improve the quality of powders, decrease the reaction temperature and the total time of the process.

Oxalate thermal decomposition is a method largely applied for AnO_2_ production, being appropriate for quantitative separation and recycling of actinides, as well as for fuel production or reprocessing of spent fuel.^7,8^ Such powders are difficult to sinter by conventional routes^9^ because of their platelet shape.

The hydrothermal decomposition (called also “decomposition under hot compressed water”) of actinide oxalates has been recently proposed by our group as an innovative approach for the safe and secure synthesis of oxide fuel.^10^ AnO_2_ (An = Th, U, Np, Pu) end-members have been prepared by this method.^10–14^ Associated U_1−*x*_An_*x*_O_2_ mixed oxides (An = Th, Pu) were obtained as well by using this method.^15,16^ Such powders are typically composed of crystallites of 10 nm or smaller, softly agglomerated at a submicrometric scale due to the low temperature of the decomposition.^11^

During our extensive studies on such compounds, we have noticed the divergent decomposition behaviour of the tetravalent plutonium and cerium oxalates.^11^ Previous results indicate that the hydrothermal decomposition of the plutonium oxalate hexahydrate leads to formation of PuO_2_ nano crystals. On the other hand, decomposition of cerium oxalate gives inconclusive results in the 60–350 °C temperature range. The product of the cerium oxalate decomposition at 400 °C/250 bar is the hexagonal Ce(CO_3_)(OH), in which cerium has a trivalent oxidation state. Keeping in mind the analogy between Ce(iii) and Am(iii), similar behaviour was expected for the decomposition of americium oxalate. On the other hand, work on (U,Pu)-mixed oxides show that the americium contained in the PuO_2_ source is fully incorporated in the oxide nanoparticles (up to 1% out of the total Pu-content).^16^

The present report addresses this matter, namely the feasibility of the methodology for the production of nanocrystalline (U,Am)O_2_ and (U,Pu,Am)O_2_ solid solutions. Such systems are particularly complex due to the high oxygen potential, chemical disorder in the cationic sublattice, and significant self-irradiation effects. Thus, the stability of the solid solutions of different chemical composition and forms (powder or pellets, under- or fully stoichiometric in oxygen) under α self-irradiation is reported. Mechanical and structural characterization of the sintered products is also presented in this paper.

## 2. Experimental

*Caution! Americium-241 is a highly radioactive isotope (*t*_1/2_ = 432.8 years, specific activity of 126.8 GBq g^−1^). Moreover, the use of plutonium-239 (as the main component of the Pu-source) imply additional radiological hazard (*t*_1/2_ = 24 110 years, specific activity of 2.8 GBq g^−1^). Natural uranium is only weakly radioactive. All work presented in this paper has been carried out in radiological laboratories licensed for handling actinides, equipped with radiation shielding and remote handling tools.*

### 2.1 Sample preparation and mechanical processing

For the synthesis of (U,Am)O_2_ and (U,Pu,Am)O_2_ nanopowders we have used the method of hydrothermal decomposition of mixed oxalates, as originally described in Walter *et al.*^10^ in addition, cerium was used as surrogate in order to check whether it can properly simulate the Am-behaviour in (U,Am)O_2_ nanopowders over the all compositional range.

U(iv) aqueous solution was obtained by electroreduction of UO_2_(NO_3_)_2_ solution in HNO_3_ (4 mol L^−1^) containing 0.5 mol L^−1^ of hydrazine. The Pu(iv) solution was produced by dissolution of PuO_2_ in HNO_3_ (8 mol L^−1^). The Am(iii) solution was obtained by dissolution of AmO_2_ in HNO_3_ (6 mol L^−1^), while the Ce(iii) solution by direct dissolution of Ce(NO_3_)_3_·6H_2_O (Sigma, 99.99%) in milli-Q water. The metal concentration in all these solutions was of about 0.5 mol L^−1^.

Then, the nitrate actinide solutions were mixed in the desired molar ratio. The mixed solution was precipitated with excess (10–20%) of oxalic acid (aqueous solution of 0.5 mol L^−1^). The readily formed precipitate was separated from the acidic solution and washed repeatedly with milli-Q water until pH = 7. The oxalate precipitate (about 1.5–2.0 g), together with 6.5 mL of milli-Q water, was treated hydrothermally for 3.5 h at 220 °C and autogenic pressure (estimated to 25–35 bar). The process was conducted in an autoclave made out of stainless steel and containing a Teflon inset of 20 mL (Fig. S1†). The final product was washed with water, ethanol and acetone.

**Fig. 1:**
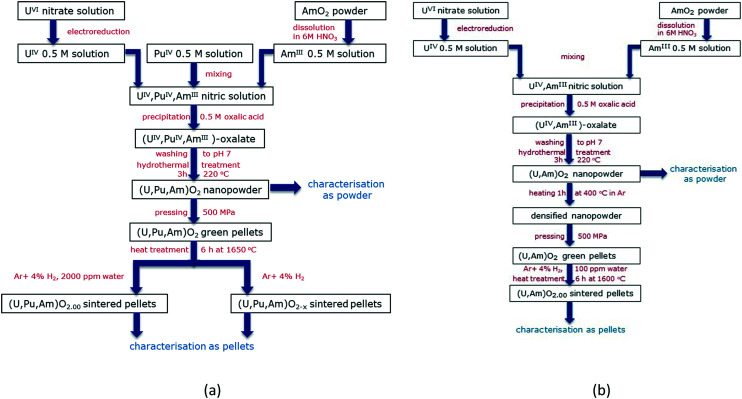
Flowcharts indicating the steps used for the production and conditioning of (U,Pu,Am)O_2_ (a) and (U,Am)O_2_ (b) solid solutions.

The first experimental trial was performed in order to produce (U,Pu,Am)O_2_ nanopowders and sintered pellets (Fig. 1a). Thus, the nc-powder was pressed uniaxially at 500 MPa to green discs. Some discs were placed into a molybdenum crucible and sintered for 6 h at 1650 °C under an atmosphere of Ar/H_2_ (4%) and about 2000 ppm of moisturizing water (heating and cooling ramps of 200 °C h^−1^). Other disks were sintered for 6 h at 1650 °C under an atmosphere of dry Ar/H_2_ (4%).

Further experiments were achieved in order to obtain (U,Am)O_2_ solid solutions. Since the process used for the production of the (U,Pu,Am)O_2_ pellets did not result in very high density disks (please see the Results and discussions section), an additional heating stage (1 h at 400 °C under Ar) was introduced in the workflow (Fig. 1b). In this case, the sintering has been performed only under one condition (6 h at 1600 °C under an atmosphere of Ar/H_2_ (4%) and about 100 ppm of moisturizing water, heating and cooling ramps of 200 °C h^−1^).

In order to check the limit of the incorporation of trivalent Am-cations in the fluorite structure, additional experiments were performed using cerium as americium substitute. U(iv) and Ce(iii) solutions were mixed in molar ratios of 90 : 10, 80 : 20, 70 : 30, 60 : 40, 50 : 50, 40 : 60, 25 : 75 and 0 : 100. All the other procedural steps were the same as described for the synthesis of (U,Am)O_2_.

### 2.2 Characterization

#### 2.2.1 Chemical and isotopic characterization

Uranium concentrations were determined by isotope dilution thermal ionization mass spectrometry and ^241^Am mass by calorimetry. The uranium isotopic composition was determined by total evaporation thermal ionization mass spectroscopy (TE-TIMS) and the plutonium isotopic composition by TIMS & alpha spectrometry (for ^238^Pu).^17–20^ The neptunium content was extrapolated from the chemical analysis of the parent Am-source.

#### 2.2.2 Powder X-ray diffraction

The powder XRD measurements were performed by using a Bruker D8 diffractometer mounted in a Bragg–Brentano configuration with a curved Ge (1,1,1) monochromator and a ceramic copper tube (40 kV, 40 mA) and supplied with a LinxEye position sensitive detector. The data were collected by step scanning in the angle range 10° ≤ 2*θ* ≤ 120° with a step size of 0.02° (2*θ*); total measuring time was about 5 h. Refinement of the data were done with Jana 2006 software.^21^

In order to avoid any dispersion of radioactive powders into the glovebox, the measurements were performed on about 10 mg of powder immobilized in a bi-component epoxy resin on a sample holder. (Fig. S3†). The measurements were repeated over a two-years timeframe so as to follow the swelling of the oxides as a function of time/radiation dose.

#### 2.2.3 Microscopy

Transmission electron microscopy analyses were performed on a TecnaiG2 (FEI™) 200 kV TEM modified during its construction to enable the examination of radioactive samples. The microscope is equipped with field emission gun, a Gatan™ Tridiem GIF camera, an electron energy-loss spectrometry (EELS) analysis system, and a high-angle annular dark-field (HAADF) detector for the scanning transmission electron microscopy (STEM) imaging.

The scanning electron microscope (SEM) used in this work was a Philips XL40 which has the column, chamber and high voltage power supply placed in a glovebox.

#### 2.2.4 High-resolution XANES experiments

For the U_0.80_Am_0.20_O_2_ materials, U M_4_ and Am M_5_ edge high energy resolution X-ray absorption near edge structure (HR-XANES) spectroscopy technique was performed at the CAT-ACT-beamline for catalysis and actinide research (hereafter CAT-ACT beamline) of the KIT synchrotron light source facility, Karlsruhe, Germany.^22^ Spectra acquisition was done utilising a Johann type X-ray emission spectrometer. The incident beam was monochromatized by a Si (111) double crystal monochromator (DCM) and focused and subsequently narrowed down by slits onto the sample to a spot size of about 200 μm × 200 μm. The X-ray emission spectrometer consists of four Si (220) crystals with 1 m bending radius and a single diode VITUS silicon drift detector (Ketek, Germany), which together with the sample are arranged in a vertical Rowland circle geometry.

UO_2_ and AmO_2_ were used as reference to calibrate the respective HR-XANES spectra. The main absorption maximum was set to 3275.5 keV and 3890.8 keV for UO_2_ and AmO_2_ respectively.^23^ The sample cells were placed into a double-containment multi-position cell, where the inner compartment was sealed by 8 μm and the outer compartment by 13 μm Kapton foil, respectively (Fig. S3†). The experimental energy resolution during the U M_4_ and Am M_5_ HR-XANES measurements was estimated to be 1 eV and 1.3 eV, respectively. The HR-XANES spectra were measured with step size 0.1 eV from −10 eV to +25 eV from the white line (WL) of the respective edge and 0.5 eV in all other parts of the spectra. At least two spectra were averaged for each sample. The sample, crystals, and detector were enclosed in a box filled with helium to minimize intensity losses due to scattering and absorption of photons in air. A constant helium flow was maintained to keep the oxygen level below 0.1%. No effect of radiation damage in the materials was evident during the measurements.

We estimated the relation between U(iv) and U(v) by (i) assuming that the main absorption maxima of U(iv) and U(v) have the same absorption intensity for 100% U(iv) or U(v) present in a sample; note that the main peak of the spectrum of U(v) will have higher intensity than U(iv) as a result of less electrons in the *f* states thus the U(v) contribution will be underestimated; (ii) only mixtures of U(iv) and U(v) are present in the two samples studied. Generally, the main peak intensity does not necessarily strictly follow the absorption cross section. But because U(v) and U(iv) occupy the same crystallographic positions and have very similar electronic structure, the approach is justified. The trend in intensity change will follow the change of electron density in the *f* states, *i.e.* the uranium oxidation state change. It should be also mentioned that the HR-XANES spectrum is a cut through a resonant inelastic X-ray scattering map and thus the intensity of the U(v) peak is additionally influenced.^23^ We then calculated the ratio U(iv)/U(v) characteristic peak intensities for the as prepared and sintered samples.

## 3. Results and discussion

### 3.1 Chemical characterization

The chemical composition of the synthetized compounds obtained on the basis of the chemical characterization is presented in Table 1. The uranium and plutonium isotopic compositions are reported in the Table S2 as a ESI.†

**Table 1:** Chemical composition and crystallographic data of the nanocrystalline mixed oxides

Target composition	Actual composition	*a*, Å	Crystal size, nm	Strain
U_0.75_Pu_0.20_Am_0.05_O_2_	U_0.769_Pu_0.171_Am_0.056_Np_0.004_O_2_	5.451(1)	13 ± 2	22 × 10^−4^
U_0.90_Am_0.10_O_2_	U_0.878_Am_0.114_Np_0.008_O_2_	5.466(1)	12 ± 2	20 × 10^−4^
U_0.80_Am_0.20_O_2_	U_0.761_Am_0.223_Np_0.016_O_2_	5.466(1)	10 ± 2	31 × 10^−4^

Due to the ageing process, the americium contains about 7% ^237^Np, and the plutonium about 2% ^241^Am. It can be observed that, despite inherent uncertainties in the composition of the reagents (moisture content, presence of the decay products), the final compositions are very close to the targeted ones. For simplification, we will use further the target composition.

### 3.2 Dimensional measurements

The sintered disks were measured using a micrometer screw gauge (for the diameters) and a dial indicator (for the heights). The weights were measured using a calibrated Sartorius precision balance. The results are summarized in Table S1.† The (U,Am)O_2_ disks showed geometrical densities between 83% TD (theoretical density) and 96% TD, as function of the composition. However, hydrostatic measurements performed on selected samples result in values that are about 3% higher, explained by the important geometrical deviations due to the axial shrinkage. In a similar manner, the (U,Pu,Am)O_2_ disks sintered under Ar/H_2_ + 2000 ppm H_2_O indicated geometrical densities between 72% TD and 79% TD, while the (U,Pu,Am)O_2_ disks sintered under dry Ar/H_2_ atmosphere between 83% TD and 87% TD.

### 3.3 Powder XRD and morphological characterization of the freshly synthesized specimens

#### 3.3.1 (U,Pu,Am)O_2_ samples

**Fig. 2:**
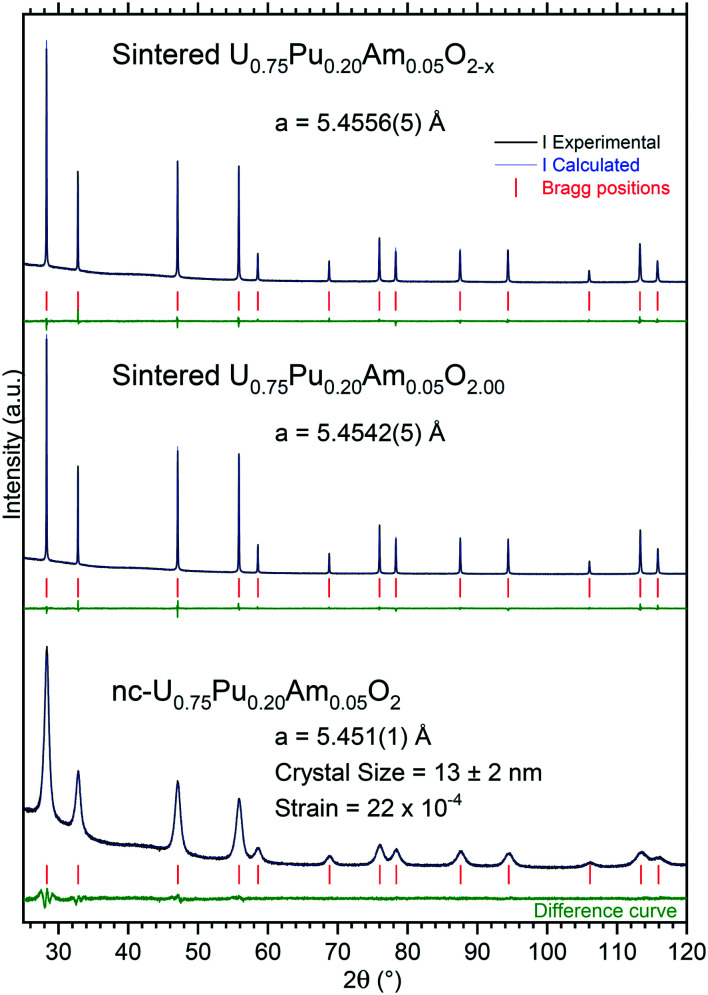
XRD pattern of the nanocrystalline product (nc-U_0.75_Pu_0.20_Am_0.05_O_2_), U_0.75_Pu_0.20_Am_0.05_O_2.00_ sintered under moisturized Ar/H_2_, and U_0.75_Pu_0.20_Am_0.05_O_2−*x*_ sintered under dry Ar/H_2_, recorded instantly after synthesis/ conditioning.

The nano-crystalline product (nc-U_0.75_Pu_0.20_Am_0.05_O_2_) obtained by hydrothermal decomposition of the oxalate was characterized by powder X-ray diffraction (Fig. 2). The lattice parameter obtained is in good agreement with the expected value assuming Vegard's law. The broad diffraction peaks obtained for this material is characteristic of nanocrystalline powder, and the Williamson–Hall plot^24^ gave a crystal size value of 13 ± 2 nm. The sample was single phase, and diffraction peaks were symmetric, suggesting a good homogeneity of the specimen.

Under moisturized Ar/H_2_ sintering atmosphere, the material was expected to be stoichiometric U_0.75_Pu_0.20_Am_0.05_O_2.00_. The lattice parameter obtained in this case was 5.4542(5) Å. Under dry Ar/H_2_ sintering atmosphere, the lattice parameter obtained was equal to 5.4556(5) Å. This higher value compared to the former one is in agreement with the formation of sub-stoichiometric oxide U_0.75_Pu_0.20_Am_0.05_O_2−*x*_. However, the lattice parameter difference was very small between the two variations, suggesting a very limited deviation from stoichiometry, with an overall O/M composition of about 1.995 when compared to the U–Pu–O system.^25^

**Fig. 3:**
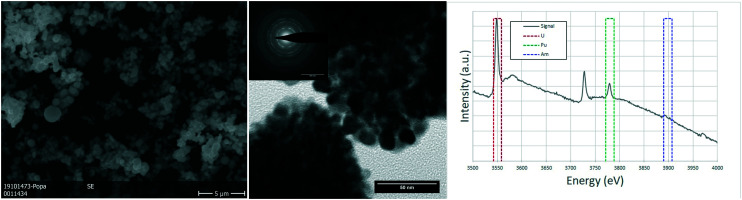
nc-U_0.75_Pu_0.20_Am_0.05_O_2_: detail of the (U,Pu,Am)O_2_ nanocrystals forming the agglomerates in a SEM image (left). TEM image showing two agglomerations of nanoparticles, with the corresponding polycrystalline electron diffraction (center). The EELS spectrum showing the white lines of the M_4_ and M_5_ edges for both uranium and plutonium and the M_5_ edge for americium, together with the integration areas used for the semi-quantification (right).

Due to the size of the nanoparticles in the original powder, the SEM cannot resolve them individually, and only the agglomerates were observed in the micrographs of Fig. 3, with their characteristic round morphology and sizes ranging from <500 nm to 2 μm. TEM analyses shed more light on the morphology and composition. The agglomerates that could be observed in the SEM micrographs were clearly distinguishable and made out of smaller nanoparticles, with sizes of 5–20 nm. Electron diffraction patterns obtained from these agglomerates showed the FCC structure of the nanoparticles, compatible with the lattice parameter calculated from XRD. EELS analyses proved the composition of the nanocrystallites to be close to the original composition, obtaining results with slight variations around 75–80% at. U, 18–20% at. Pu and about 5% at. Am.

**Fig. 4:**
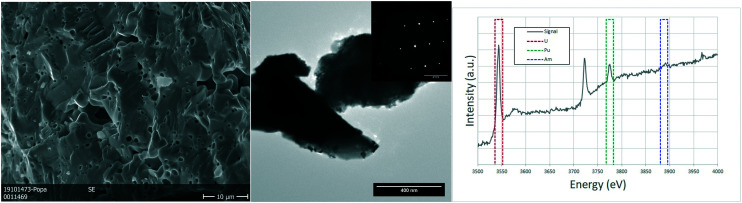
Sintered U_0.75_Pu_0.20_Am_0.05_O_2.00_: detailed SEM image on the porosity of the sample (left). TEM image showing a typical view of the sample with the electron diffraction illustrating a single crystal (center). EELS spectrum showing the edges for uranium and plutonium and the areas used to estimate the ratios between U, Am and Pu (right).

The analysed specimen of stoichiometric U_0.75_Pu_0.20_Am_0.05_O_2.00_ pellet showed a large fraction of pores homogeneously distributed over the whole sample, in line with the geometrical density measurements which showed 72–79% TD (see Table S1†). The scanning electron microscopy revealed no further information on this sample, as the examples in the Fig. 4. TEM analyses showed micrometric grains with no defects or inhomogeneities. The semi-quantitative analyses showed the ratio between the actinides to be in good agreement with those of the fabrication. The structure seems to be preserved as FCC as proven by the electron diffractions obtained on several grains.

**Fig. 5:**
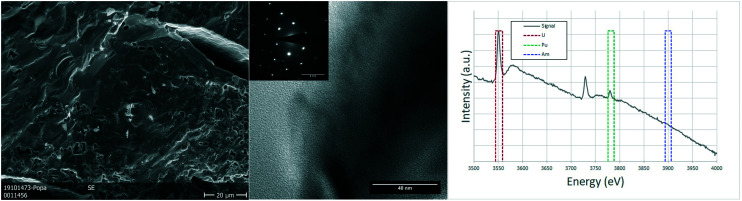
Sintered U_0.75_Pu_0.20_Am_0.05_O_2−*x*_: SEM micrograph showing the variations on the porosity in different regions of the sample (left). TEM image revealing no defects (center) and electron diffraction showing the monocrystalline nature of the region, coherent with a [1 1 1] orientation of a FCC structure (inset). EELS spectrum of the same region used for the semi-quantification of the actinides (right).

In the case of the oxygen sub-stoichiometric specimen (U_0.75_Pu_0.20_Am_0.05_O_2−*x*_), the porosity seemed to be on average lower than that of the stoichiometric sample (in line with the geometrical density measurements, 83–87% TD). However, some regions showed an increased number and size of pores (Fig. 5), which led to the creation of a channelling effect, connecting large pores into continuous channels separating individual grains. The TEM study showed once again no defects or relevant details observed on the crystals which have an FCC structure (as seen on the electron diffraction proving the [111] orientation of the crystal) and large grains.

Note that this limited direct sintering behaviour is contrasting with recent results on UO_2+*x*_ powders obtained by the same method for which a much higher density was reached,^26^ and very likely originates from the extremely high free volume in the as-synthesized material. Thus, in further trials, a new heating stage (1 h at 400 °C under argon) was introduced in the workflow (prior to pressing the powder into discs or pellets), in order to pre-densify the powders and to vaporise the water and to release the trapped gases. The result of this stage was beneficial, as it can be observed from the data summarised in Table S1.†

#### 3.3.2 (U,Am)O_2_ samples

**Fig. 6:**
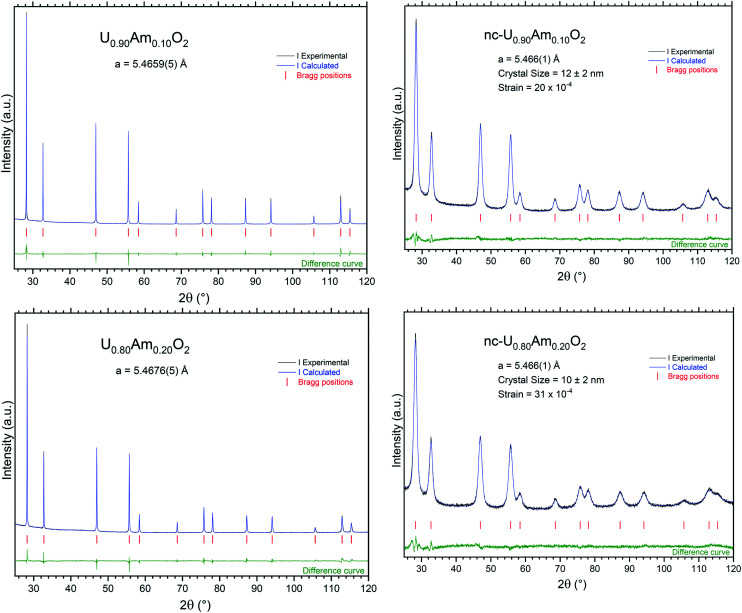
XRD patterns of the nanocrystalline and sintered (U,Am)O_2_ specimens recorded instantly after synthesis/conditioning.

The nanocrystalline products (nc-U_0.90_Am_0.10_O_2_ and nc-U_0.80_Am_0.20_O_2_) obtained by hydrothermal decomposition of the corresponding oxalates in autoclave were characterised by XRD (Fig. 6). The lattice parameters obtained (5.466(1) Å for both nc-U_0.90_Am_0.10_O_2_ and nc-U_0.80_Am_0.20_O_2_) are in good agreement with those expected for such an actinide composition. The broad diffraction peaks obtained for these materials are characteristic of nanocrystalline compounds, and the Williamson–Hall plot gave crystal size values of 12 ± 2 nm (nc-U_0.90_Am_0.10_O_2_), respectively 10 ± 2 nm (nc-U_0.80_Am_0.20_O_2_). The samples are monophasic, and diffraction peaks are symmetric, suggesting a good homogeneity of the specimen.

As in the previous case, the sintering process induced a sharpening of the diffraction peaks in the XRD measurements due to crystal growth. Under moisturised Ar/H_2_ sintering atmosphere, the materials are expected to be stoichiometric U_1−*x*_Am_*x*_O_2.00_. The lattice parameters obtained in this case were 5.4659(5) Å for U_0.90_Am_0.10_O_2.00_ and 5.4676(5) Å for U_0.80_Am_0.20_O_2.00_, respectively.

**Fig. 7:**
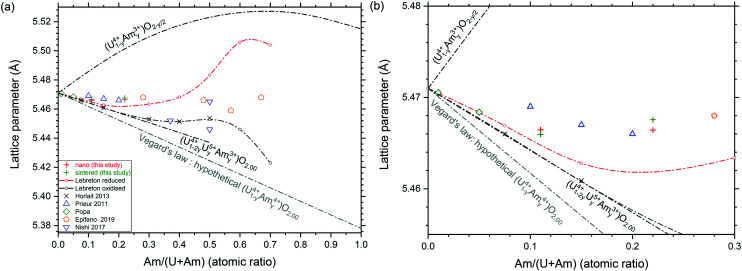
Variation of lattice parameters in the (U,Am)O_2(−*δ*)_ system: full range of composition (a) and closer view of the compositions of interest (b).

Lattice parameter of the (U,Am)O_2_ mixed oxides are difficult to interpret due to the occurrence of complex charge distribution in this system.^27,28^ Lattice parameters of nanocrystalline mixed oxides and sintered specimens obtained in this study are presented in Fig. 7 together with other experimental values present in the literature,^27–31^ and models proposed by Nishi *et al.*^31^ the expected trend for fully reduced material (U^4+^_1−*y*_Am^3+^_*y*_)O_2−*y*/2_ containing only uranium at the oxidation state IV and americium at the oxidation state III is presented by the curve at the top of the graph. This trend was experimentally observed in different mixed oxides.^32–34^ It corresponds to the progressive change from the tetravalent fluorite M^4+^O_2_ dioxide to trivalent bixbyite-like M^3+^_2_O_3_ sesquioxide end-members. However, it seems that this level of reduction is never reached for (U,Am)O_2_ mixed oxides, even after reductive sintering. The curve resulting from Vegard's law between the UO_2.00_ to AmO_2.00_ end-members is shown at the bottom part of the graphs. However, it was well established that the Vegard's law is irrelevant for this system due to charge transfer occurring between uranium and americium in the mixed oxide.^27,28,31^ In stoichiometric mixed (U,Am)O_2.00_ oxides, Am(iii) and U(v) were reported to be present in equivalent proportions.^28^ Since (U^4+^_1−2*y*_U^5+^_*y*_Am^3+^_*y*_)O_2.00_ has in average larger ionic radii than the hypothetical (U^4+^_1−*y*_Am^4+^_*y*_)O_2.00_ with the same U/Am composition, lattice parameters of stoichiometric mixed oxides (U,Am)O_2.00_ show systematically larger lattice parameters than those suggested by Vegard's law. Experimental values obtained by Lebreton^29^ are also reported in Fig. 7 with the higher values corresponding to mixed oxides freshly reduced material (1 hour, 1100 °C, Ar/H_2_ (4%)), and the lower values corresponding the same product after spontaneous oxidation under storage condition (room temperature, air) occurring from few hours to few weeks after reductive treatment. This shows that at high Am content, a broad range of lattice parameters (*i.e.* O/M ratios) can be achieved but with a high sensitivity to oxidation, while at low Am content, mixed oxide seems close to stoichiometry from fresh reduction to long air storage. From the results presented in Fig. 7, the lattice parameters for the materials in this work suggest a composition close to stoichiometry (O/M≈2) for U_0.90_Am_0.10_O_2_ while U_0.80_Am_0.20_O_2_ seems to have a significantly higher lattice parameter than the expected trend, which suggest a significant substoichiometry (O/M < 2).

We have checked the limit of the Ce(iii) incorporation (as a surrogate for Am(iii)) in the (U,Ce)O_2_ solid solutions by using the hydrothermal decomposition of mixed oxalates method over the full compositional range, *i.e.* to answer the question until which cerium concentration we can apply this synthesis route without the formation of undesired micro-crystalline Ce(CO_3_)OH as observed in pure cerium oxalate hydrothermal decomposition.^11^ The results showed that the nanocrystalline solid solutions FCC structure formed up to a composition of U_0.5_Ce_0.5_O_2_, which indicates that this method might be applied for materials with an americium content up to 50%; above this concentration, the charge compensation needs further oxidation of uranium into hexavalent form. Note that nanocrystalline solid solutions with FCC structure can form over the full compositional range in the uranium–cerium system by using an alternative precursor^36^ (hydrothermal treatment of mixed hydroxides).

### 3.4 Cationic charge distribution in sintered and nc-U_0.80_Am_0.20_O_2_

**Fig. 8:**
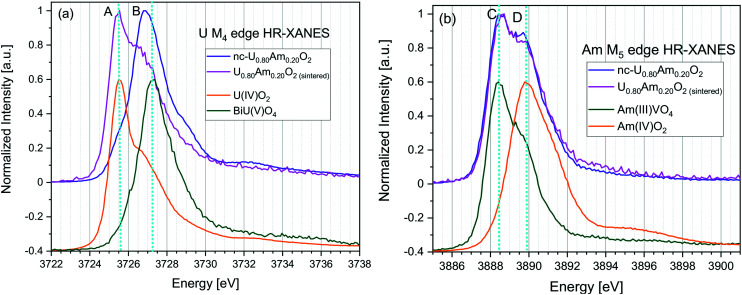
HR-XANES spectra acquired at U M_4_ (a) and Am M_5_ (b) edges for nc-U_0.80_Am_0.20_O_2_, nc-U_0.80_Am_0.20_O_2_ after sintering and UO_2_, BiUO_4_, AmVO_4_, AmO_2_ reference materials.

HR-XANES spectroscopy data at the U M_4_ edge for as-synthesised nc-U_0.80_Am_0.20_O_2_ revealed the presence of mixed U(iv) and U(v) oxidation states, with majority of the U atoms accommodating U(v) oxidation state. The energy position of feature *B* in the U^V^BiO_4_ spectrum^37^ located at about 3727.3 eV is characteristic of the main absorption intensity of U(v) (*cf.*Fig. 8a).^16,35^ Note that the time from synthesis to measurement was 6 months for the nc-U_0.80_Am_0.20_O_2_. As previously described for (U,Ce)O_2_ nanoparticles,^36^ the oxidation of the material occurs under storage conditions due to the high sensitivity for oxidation of these materials due to their high specific surface area. Given the size of the nanocrystals (10 ± 2 nm) in nc-U_0.80_Am_0.20_O_2_, and the storage condition (nitrogen atmosphere with up to 1% O_2_), it can be expected that this majority of U(v) in the nanocrystalline samples is not the result of the synthesis route, but the result of the oxidation of the material before the HR-XANES measurement. We reported such oxidation also previously for U_*x*_Pu_1−*x*_O_2_ nanosized powders.^16^ A small amount of U(v) in the bulk UO_2_ reference is due to oxidation as UO_2_ was kept in air. U M_4_ edge HR-XANES data acquired 76–78 days after sintering clearly indicate reduction of uranium toward U(iv) for sintered material. The post-edge region of the spectra exhibits two broad peaks typical for the FCC cubic structure (see Fig. S4†). Prior to sintering these shape-resonances in the range from 3740 eV to 3770 eV have rather low intensity, whereas after sintering those gain intensity and are better resolved. This result is in accordance with the increased particle size and crystallinity reported by XRD. Similar trend has been reported earlier for nanosized and bulk PuO_2_.^38^

The Am M_5_ edge spectra of nc-U_0.80_Am_0.20_O_2_ and the sintered U_0.80_Am_0.20_O_2_ are depicted in Fig. 8b. The energy positions of the first intense peaks and the main peak of the Am(iii) reference coincide (line C in Fig. 8b). This suggests that Am(iii) dominates in both samples and is in agreement with previous reports that Am(iii) stabilizes along with U(v). It is evident that the shoulder of the spectrum is positioned at the energy position of the main spectral maximum of Am^IV^O_2_ (line D). This shoulder is also present for Am(iii) in AmVO_4_ thus its existence does not necessary indicate Am(iv) in the two samples. However, after sintering the intensity of the shoulder decreases and this suggests reduction of Am(iv) to Am(iii). This result is an evidence for minor amount of Am(iv) stabilized in the as prepared nc-U_0.80_Am_0.20_O_2_ crystals. The presence of Am(iv) in the nanomaterial nc-U_0.80_Am_0.20_O_2_ with 20% Am content is a new finding since americium is found to be in its trivalent oxidation state in the U_1−*x*_Am_*x*_O_2±*y*_ (*x* = 0 to 0.5) bulk system in previous studies,^27–31^ even after calcination under air.^39,40^ We assume that Am is partially oxidised on the surface of the nanoparticles.

It must be noted that, despite this new observation suggesting a minor amount of Am(iv) in nanosized material, the overall valence composition of uranium and americium in the nanoparticles is very similar to the one reported for U_1−*x*_Am_*x*_O_2±*y*_ sintered materials oxidized in air up to 1200 °C by Epifano *et al.*^39,40^ for similar U/Am compositions. However, the results of these authors suggests that, for this degree of oxidation, the cubic phase of U_0.80_Am_0.20_O_2±*y*_ should have a lattice parameter of about 5.433 Å, which is very much smaller than the value of 5.466(1) Å measured in our case on nc-U_0.80_Am_0.20_O_2_ soon after synthesis. This result tends to confirm that the oxidation of the nanocrystals occurred under storage conditions before the HR-XANES measurement, due to the very high sensitivity of such materials against oxidation, as it was observed previously.^36^

### 3.5 Lattice expansion of (U,Pu,Am)O_2_ and (U,Am)O_2_

**Fig. 9:**
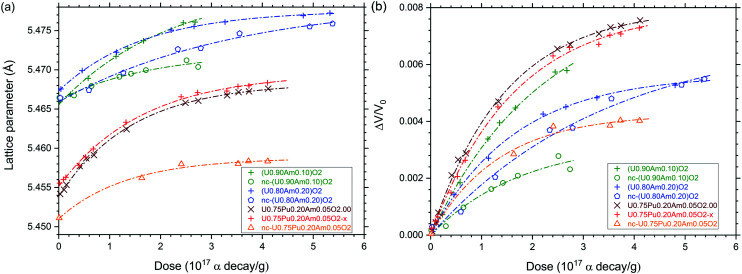
Variation of the lattice parameter (a) and relative lattice volume (b) of (U,Pu,Am)O_2_ (nano), sub- and stoichiometric and (U, Am)O_2_ 10 and 20%, nano and stoichiometric as function of the alpha dose.

The behaviour of the different mixed oxides under self-irradiation was monitored by XRD (Fig. 9). The expansion of lattice parameters follows an exponential trend with the equation:

1 *a* = *a*_0_ + Δ*a*(max) × [1 − exp(−*D*/*B*)]

where *a* is the lattice parameter in Å, *a*_0_ correspond to the lattice parameter (in Å) free from damages, Δ*a*(max) is the maximal lattice expansion compare to *a*_0_ (in Å), and *D* is the dose accumulated in the material (in α/g) and *B* is the specific dose out of the exponential trend (in α/g). The lattice volume variation of the material can be described as follows:

2 Δ*V*/*V*_0_ = *A* × [1 − exp(−*D*/*B*)]

where Δ*V*/*V*_0_ is the relative lattice volume variation, *A* correspond to the maximal volume expansion under self-irradiation.

Lattice parameter values and lattice volume expansion presented in Fig. 9 were fitted to eqn (1) and (2) in order to obtain the constants *a*_0_, Δ*a*(max), *B* and *A* (Table 2). One can see that the specific dose of the exponential trend (*B*) was about 2 × 10^17^ α/g and the maximal volume swelling was about 0.8% at saturation, which is very similar to what was already described for pure PuO_2_,^41^ AmO_2_,^42^ (U,Pu)O_2_ (ref. 43) or (U,Am)O_2_ (ref. 30) mixed oxides. In case of all nano-crystalline materials and of sintered U_0.80_Am_0.20_O_2_, the volume swelling at saturation was significantly lower. For the nano-crystalline materials, this may be explained by the fact that alpha irradiation is causing disorder to phases which are already significantly disordered compared to sintered specimens, or by the shorter diffusion path of alpha particles to the grain boundaries. In case of the U_0.80_Am_0.20_O_2_ sintered sample, the low swelling might be explained by a significant substoichiometry (O/M < 2), as suggested by the initially large lattice parameter, in combination with a moderate swelling under alpha self-irradiation. Alternatively, the lattice variation observed could result from the superposition of damage accumulation (volume increase) and oxidation (volume decrease). However, oxidation is not the privileged explanation here, since (i) the typical exponential trend observed during the time of measurement would suggest that oxidation and alpha damage swelling occurred along similar time frame which seems unlikely, and (ii) a strong oxidation of the nc-U_0.80_Am_0.20_O_2_ as observed with HR-XANES would bring much more pronounced lattice shrinkage down to about 5.433 Å according to the work of Epifano *et al.*^39,40^ therefore, the epoxy resin used for XRD sample preparation has probably a protective effect against oxidation.

**Table 2:** Fit of *a*_0_, Δ*a*(max), *B* and *A* constants in eqn (1) and (2)

	*a* = *a*_0_ + Δ*a*(max) × [1 – exp(−*D*/*B*)]			*A*, %
	Δ*V*/*V*_0_ = *A*[1 – exp(−*D*/*B*)]			
	*a*_0_ (Å)	Δ*a*(max) (Å)	*B* (α/g)	
U_0.75_Pu_0.20_Am_0.05_O_2.00_	5.4539	0.014	1.46 × 10^17^	0.80
U_0.75_Pu_0.20_Am_0.05_O_2−*x*_	5.4551	0.014	1.64 × 10^17^	0.80
nc*-*U_0.75_Pu_0.20_Am_0.05_O_2_	5.4510	0.008	1.33 × 10^17^	0.43
U_0.90_Am_0.10_O_2_	5.4656	0.015	2.18 × 10^17^	0.84
nc*-*U_0.90_Am_0.10_O_2_	5.4662	0.006	1.81 × 10^17^	0.33
U_0.80_Am_0.20_O_2_	5.4673	0.010	1.65 × 10^17^	0.56
nc*-*U_0.80_Am_0.20_O_2_	5.4659	0.013	3.45 × 10^17^	0.71

Note that the effect of alpha self-irradiation on particle size and strain of the nanocrystalline materials has also been followed. However, none of the variation observed seems significant (Table S3 and Fig. S5†).

## 4. Conclusions

We present here the first synthesis of homogeneous, nanocrystalline (U,Am)O_2_ and (U,Pu,Am)O_2_ solid solutions. The results confirm the feasibility of the oxalate decomposition under hot compressed water method for production of Am-containing oxides. Experiments performed using cerium surrogate indicates that nanocrystalline solid solutions with FCC structure is forming up to *x* = 0.5 in U_1−*x*_Ce_*x*_O_2_, suggesting that this method might be extended up to an americium content of 50%. This demonstrates that this practice can be applied for synthesis of pure tetravalent actinide oxides (as in the cases of Th, U, Np and Pu) and mixed oxides (as demonstrated for U–Th, U–Pu, U–Am and U–Pu–Am oxide systems). Conventional sintering was tested and gives promising results, especially by introducing of pre-densification heating stage in the workflow. Both uranium and americium appear in mixed oxidation states in the as-prepared and sintered U_0.80_Am_0.20_O_2_ nanocrystals but the fluorite FCC structure is preserved. Uranium is mostly oxidised to its pentavalent form and Am(iii) is mainly present in the U_0.80_Am_0.20_O_2_ nanocrystals, but large amount of U(v) is reducing to U(iv) during sintering. We show that part of Am is in tetravalent form in the as-prepared U_0.80_Am_0.20_O_2_ nanocrystals.

## Author contributions

J.-F. V. conceptualisation; formal analysis (XRD); investigation (XRD); methodology; validation; writing – original draft; writing – review & editing. D. F. project administration; resources; writing – review & editing. O. W. methodology; writing – original draft. O. D. B. investigation (SEM, TEM); writing – original draft. D. B. investigation (XRD); writing – original draft. E. Z. supervision (chemical analysis); writing – original draft. N. P. formal analysis (XANES); investigation (XANES); methodology (XANES); validation (XANES); writing – original draft; writing – review & editing. T. V. formal analysis (XANES); funding acquisition (XANES); project administration (XANES); supervision (XANES); validation; writing – original draft; writing – review & editing. R. K. project administration; resources; supervision; writing – review & editing. K. P. conceptualisation; investigation (XRD); methodology; supervision; validation; writing – original draft; writing – review & editing.

## Conflicts of interest

The authors declare no conflict of interest.

## Supplementary Material

CE-024-D2CE00527A-s001
