# Supplementary material for: Synthesis and characterization of homogeneous (U,Am)O2 and (U,Pu,Am)O2 nanopowders
Source: CrystEngComm. 2022 Aug 31;24(36):6338–48. doi: 10.1039/d2ce00527a (PMC9486969; doi:10.1039/d2ce00527a)
Supplement: CE-024-D2CE00527A-s001 [file CE-024-D2CE00527A-s001.pdf]

Electronic Supplementary Material (ESI) for CrystEngComm.
This journal is © The Royal Society of Chemistry 2022

Synthesis and characterization of homogeneous $(U,Am)O_2$ and $(U,Pu,Am)O_2$ nanopowders

Jean-François Vigier,[1,*] Daniel Freis,[1] Olaf Walter,[1] Oliver Dieste Blanco,[1] Daniel Bouëxière,[1] Evelyn Zuleger,[1] Natalia Palina,[2] Tonya Vitova,[2] Rudy J.M. Konings,[1] Karin Popa,[1,**]

[1] *European Commission, Joint Research Centre, Karlsruhe, Germany*

[2] *Institute for Nuclear Waste Disposal (INE), Karlsruhe Institute of Technology, P.O. 3640, D-76021 Karlsruhe, Germany*

[*] *Corresponding author, e-mail: jean-francois.vigier@ec.europa.eu*

[**] *Corresponding author, e-mail: karin.popa@ec.europa.eu*

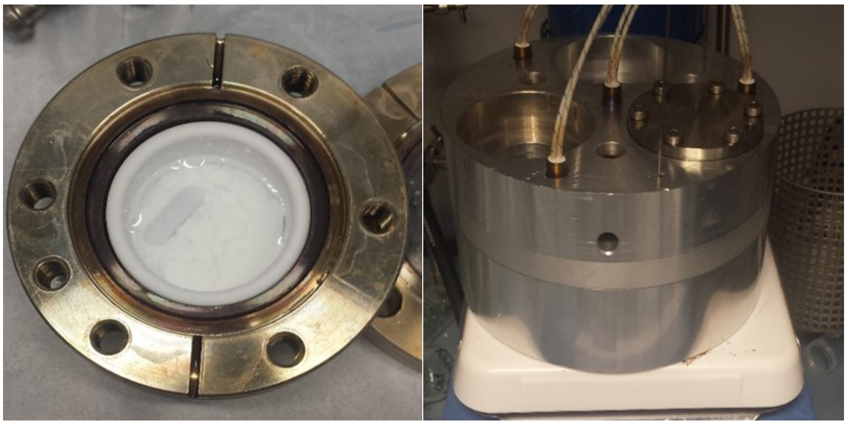

Fig. S1 The autoclave (with Teflon insets) and the heating mantle used for the present experiment

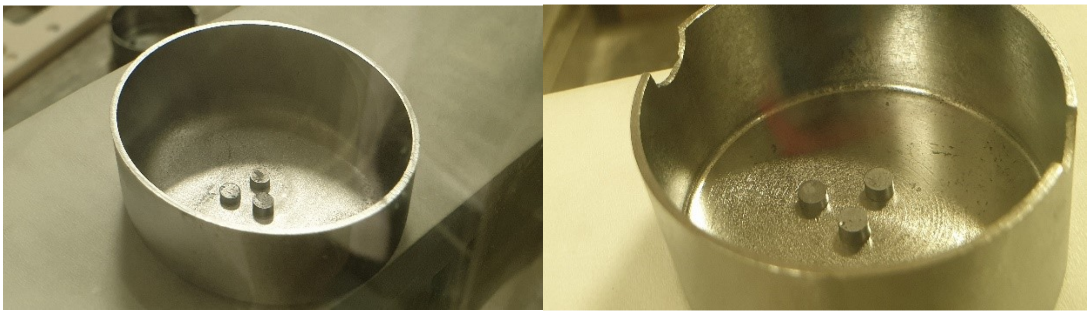

Fig. S2 Pictures of the sintered disks: $U_{0.90}Am_{0.10}O_2$ (left) and $U_{0.80}Am_{0.20}O_2$ (right)

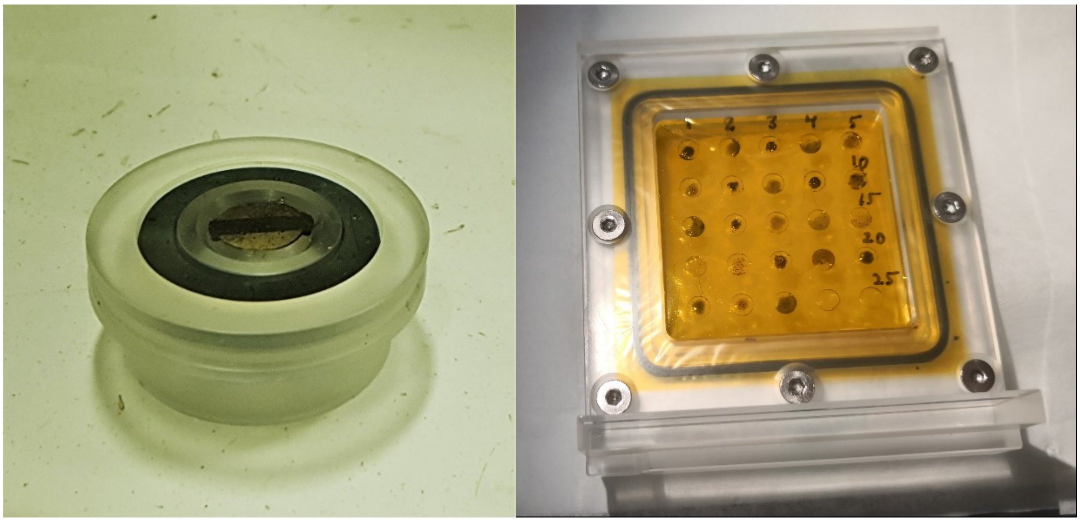

Fig. S3 Sample holders with embedded material for XRD and XAS measurements

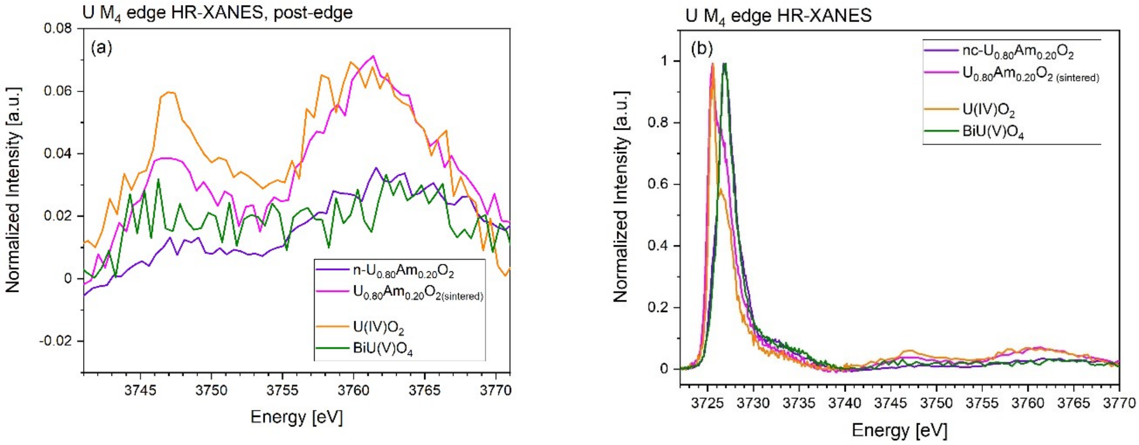

Fig. S4 HR-XANES U M_4 edge for as-synthesised nanoparticles (lilac) and sintered (magenta) $U_{0.80}Am_{0.20}O_2$ samples in the post-edge (a) and whole energy (b) range along with the $U(IV)O_2$ (orange) and the $BiU(V)O_4$ (green) references.

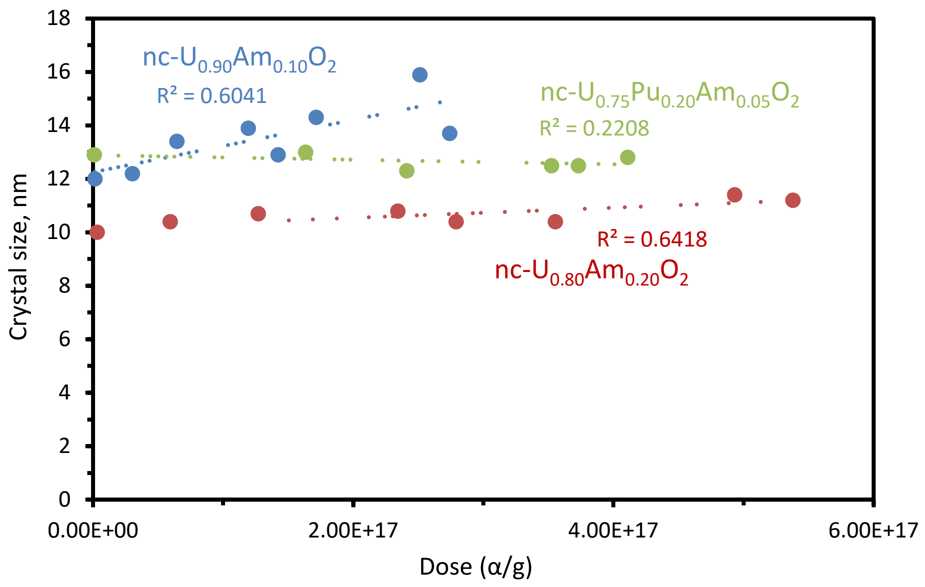

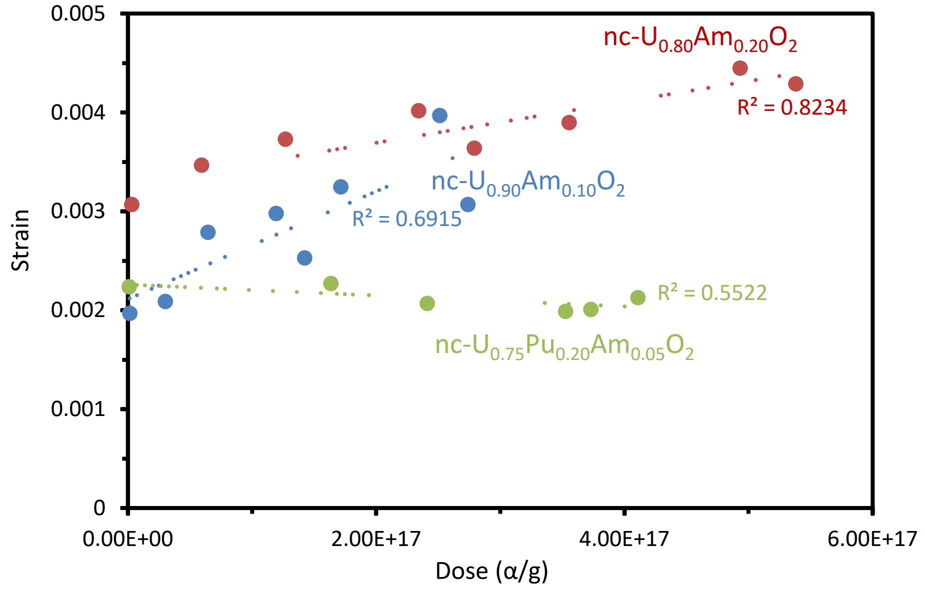

Fig. S5. Variation of crystal size (top) and strain (bottom) of nanocrystalline materials as a function on alpha dose

Table S1. Characterisation of sintered disks out of geometrical measurements (calculated for a theoretical density of 11 g/cm³)

Composition	Disk/ Pellet	h, mm	d, mm	m, mg	ρ, g/cm³	% TD		Observations
						Geometrical	Hydrostatic	
$U_{0.90}Am_{O.10}O_{2.00}$	1	1.765	3.424	189	10.382	94.64		
	2	2.230	3.611	240	10.509	95.80	98.80	
	3	1.668	3.640	166	9.564	87.18		
$U_{0.80}Am_{O.20}O_{2.00}$	1	1.628	3.73	179	10.062	91.72		[a]
	2	2.348	3.82	247	9.080	82.80		[a]
	3	2.055	3.675	222	10.185	92.94	96.04	
$U_{0.75}Pu_{0.20}Am_{0.05}O_{2-x}$	1	0.762	4.247	86	7.971	72.46		[a]
	2	1.223	4.244	150	8.674	78.86		[a]
	3	1.234	4.261	150	8.529	77.53		[a]
$U_{0.75}Pu_{0.20}Am_{0.05}O_{2.00}$	1	0.780	4.172	102	9.571	87.00		
	2	1.168	4.149	149	9.440	85.82		
	3	6.957	4.133	854	9.155	83.22		[a]

[a] anisotropic radial shrinkage

Table S2. Uranium and plutonium isotopic compositions

	Mass fraction, wt%	Uncertainty, wt% [k = 2]
$^{233}U/U$	< 0.001	-
$^{234}U/U$	0.00530	0.00052
$^{235}U/U$	0.7124	0.0022
$^{236}U/U$	< 0.001	-
$^{238}U/U$	99.2823	0.0072
$^{238}Pu/Pu$	0.01398	0.00084
$^{239}Pu/Pu$	90.713	0.018
$^{240}Pu/Pu$	9.1170	0.0048
$^{241}Pu/Pu$	0.0824	0.0049
$^{242}Pu/Pu$	0.0732	0.0060

Table S3. Variation of crystal size and strain of nanocrystalline materials as a function on alpha dose

nc-$U_{0.90}Am_{0.10}O_2$			*nc*-$U_{0.80}Am_{0.20}O_2$			*nc*-$U_{0.75}Pu_{0.20}Am_{0.05}O_2$		
Dose, α/g	CS, nm	strain	Dose, α/g	CS, nm	strain	Dose, α/g	CS, nm	strain
1.28E+15	12.0	0.00197	2.96E+15	10.0	0.00307	8.66E+14	12.9	0.00224
3.00E+16	12.2	0.00209	5.93E+16	10.4	0.00347	1.63E+17	13.0	0.00227
6.45E+16	13.4	0.00279	1.27E+17	10.7	0.00373	2.41E+17	12.3	0.00207
1.19E+17	13.9	0.00298	2.34E+17	10.8	0.00402	3.52E+17	12.5	0.00199
1.42E+17	12.9	0.00253	2.79E+17	10.4	0.00364	3.73E+17	12.5	0.00201
1.71E+17	14.3	0.00325	3.55E+17	10.4	0.0039	4.11E+17	12.8	0.00213
2.51E+17	15.9	0.00397	4.93E+17	11.4	0.00445			
2.74E+17	13.7	0.00307	5.38E+17	11.2	0.00429			
